# Sydenham's chorea - clinical and evolutive characteristics

**DOI:** 10.1590/S1516-31802002000100005

**Published:** 2002-01-02

**Authors:** Maria Teresa Ramos Ascensão Terreri, Suzana Campos Roja, Claudio Arnaldo Len, Patricia Corte Faustino, Adriana Madureira Roberto, Maria Odete Esteves Hilário

**Keywords:** Rheumatic fever, Sydenham's chorea, Recurrences, Prophylaxis, Febre reumática, Coréia de Sydenham, Recorrência, Profilaxia

## Abstract

**CONTEXT:**

During the last 12 years we have observed an increase in the frequency of Sydenham's chorea in our country. We have observed that some of our patients have presented recurrence of the chorea despite regular treatment with benzathine penicillin.

**OBJECTIVE:**

The aim of our study was to evaluate clinical and evolutive characteristics of Sydenham's chorea in a group of patients followed in our Pediatric Rheumatology Unit.

**TYPE OF STUDY:**

Retrospective study.

**SETTING:**

Section of Pediatric Rheumatology - Discipline of Allergy, Clinical Immunology and Rheumatology - Department of Pediatrics - UNIFESP - EPM.

**PARTICIPANTS:**

Two hundred and ninety patients with rheumatic fever followed between 1986 and 1999.

**METHODS:**

We reviewed the records of 290 patients with rheumatic fever followed between 1986 and 1999. All patients were diagnosed according to the revised Jones criteria (1992). We included 86 patients that presented Sydenham's chorea as one of the major criteria (one or more attacks) and evaluated their clinical and evolutive characteristics as well the treatment.

**RESULTS:**

Fifty-five patients were girls and 31 were boys. The mean age at onset was 9.7 years and mean follow-up period was 3.6 years. The 86 Sydenham's chorea patients presented 110 attacks of chorea. We observed isolated chorea in 35% of the patients, and 25 (29%) presented one or more recurrences. We included only 17 of the 25 patients for further analysis, with a total of 22 recurrences of which 14 were attacks of chorea, because it was not possible to precisely detect the interval between attacks in the other patients. The approximate interval between the attacks ranged from 4 to 96 months. In 71% of the patients there was no failure in the secondary prophylaxis with benzathine penicillin, which was performed every 3 weeks.

**CONCLUSION:**

Despite the regular use of secondary benzathine penicillin prophylaxis, children with rheumatic fever have a high risk of Sydenham's chorea recurrence.

## RESUMO

**CONTEXTO:**

Observamos um aumento na freqüência de coréia de Sydenham no nosso meio durante os últimos 12 anos. Foi verificado que alguns pacientes apresentaram recorrência da coréia apesar da profilaxia regular com penicilina benzatina.

**OBJETIVO:**

Avaliar as características clínicas e evolutivas da coréia de Sydenham em um grupo de pacientes acompanhados no nosso ambulatório de Reumatologia Pediátrica.

**TIPO DE ESTUDO:**

Estudo retrospectivo.

**LOCAL:**

Setor de Reumatologia Pediátrica, Disciplina de Alergia, Imunologia clínica e Reumatologia, Departamento de Pediatria, Universidade Federal de São Paulo/Escola Paulista de Medicina.

**PARTICIPANTES:**

290 pacientes com febre reumática seguidos no período de 1986 e 1999.

**MÉTODOS:**

Revisamos os prontuários de 290 pacientes com febre reumática seguidos no período de 1986 a 1999. Todos os pacientes tiveram o diagnóstico de febre reumática de acordo com os critérios de Jones revisados (1992). Foram incluídos 86 pacientes que apresentaram coréia de Sydenham como manifestação maior (um ou mais surtos) e avaliamos suas características clínicas e evolutivas bem como tratamento.

**RESULTADOS:**

55 pacientes eram do sexo feminino e 31 do masculino. A idade média de início da doença foi 9,7 anos e o tempo médio de evolução 3,6 anos. Os 86 pacientes com coréia de Sydenham apresentaram 110 surtos de coréia. Nós observamos coréia isolada em 35% dos pacientes e 25 (29%) apresentaram uma ou mais recorrências. Foram incluídos para o estudo apenas 17 de 25 pacientes com 22 recorrências e 14 surtos de coréia, pois não foi possível detectar o intervalo entre os surtos nos outros pacientes. O intervalo entre os surtos variou de quatro a 96 meses. Em 71% dos pacientes não ocorreu falha na profilaxia secundária com penicilina, que foi realizada a cada três semanas.

**CONCLUSÃO:**

Apesar do uso regular de profilaxia com penicilina benzatina, as crianças com febre reumática têm um maior risco de recorrência de coréia de Sydenham.

## INTRODUCTION

Sydenham's chorea is an enigmatic manifestation of rheumatic fever. Although Sydenham's chorea is rare in developed countries, we have observed an increase in its frequency in our country during the last few years.^1^

It may appear as an isolated manifestation of rheumatic fever or precede or accompany an acute attack associated with other criteria of the disease.^2-5^

The relationship between Sydenham's chorea and rheumatic fever has been the subject of considerable controversy and some aspects continue to be intriguing. Since chorea is generally a late manifestation, it is unusual to find clinical or even immunological (laboratory) evidence of a streptococcal infection, and the erythrocyte sedimentation rate is usually normal.^6^

In 1985, Berrios et al.^7^ reported that 32% of their Sydenham's chorea patients suffered a recurrence despite the penicillin prophylaxis, which raised doubts not only about the relationship with the streptococci but also, in some cases, about the rheumatic fever itself. We had also observed that some of our patients presented recurrence of chorea despite regular treatment with benzathine penicillin. Our aim was to study the clinical and evolutive characteristics of Sydenham's chorea patients and the frequency of the recurrences, as well as the relationship with regular prophylaxis.

## METHODS

A retrospective evaluation of the records of 290 rheumatic fever patients diagnosed according to the revised Jones criteria (1992)^8^ was carried out between 1986 and 1999. Patients diagnosed before 1992 were reevaluated to ascertain whether they fulfilled the 1992 Jones criteria.

Children of less than 16 years of age, with well-established chorea and a follow-up period equal to or greater than one year were included in the study. Patients with a family history of chorea, or positive antinuclear antibody, were excluded.

The epidemiological data, the clinical characteristics of the attacks (initial and recurrent) and the compliance with secondary prophylaxis (every 21 days) were analyzed. We defined recurrence as when the patient presented manifestations of chorea after a minimum period of 4 months without symptoms and medication.

The anti-streptolysin O titer had been measured in the majority of patients. It was considered altered when there was an increase of two or more titer steps in relation to the previous titer (15-day interval) or when one titer was equal to or greater than 800 IU/ml (in cases with only one measurement).

## RESULTS

Of the 290 patients with rheumatic fever, 86 (29.6%) had chorea during the first attack of the disease. There were a total of 100 chorea episodes, including recurrences. Fifty-five patients with Sydenham's chorea were female (ratio 1.7:1) and 44 non-Caucasian. The mean age at onset of symptoms was 9.7 years old and the mean follow-up period was 3.6 years (Table 1).

**Table 1:** Patients with Sydenham's chorea in the first attack and recurrences, according the gender, race, age at onset and follow-up period

*Chorea*	*N*	*Gender* *(F:M)*	*Race* *(C:NC)*	*Age at onset years (mean)*	*Follow-up period years (mean)*
***First attack***	***86***	***55:31 1.7:1***	***42:44***	***97***	***3.6***
***Recurrence***	***17***	***10:7 1:4:1***	***9:8***	***9.8***	***4.8***

***F: Female C: Caucasian***

***M: Male NC: Non-Caucasian***

Sydenham's chorea occurred in isolation in 30 patients (35%) and in association with other symptoms in 56 patients (65%), during the first episode of rheumatic fever. In the latter we observed chorea (co) and carditis (ca) in 38 patients, chorea, carditis and arthritis (ar) in 14 and chorea and arthritis in 4 patients. Of the 86 patients who presented chorea at the first attack, 25 (29%) had 32 recurrences, of which 24 were of chorea. These recurrences began after a minimum of 4 months without symptoms and without specific chorea treatment. In 8 patients (10 episodes), it was not possible to precisely detect the interval between attacks and for this reason, these were not included in the study.

We therefore included only 17 of the 25 patients for further analysis, with a total of 22 recurrences of which 14 were attacks of chorea. Within this group, 10 patients were female and 9 were Caucasian. The mean age at onset was 9.8 years old and the mean follow-up period 4.8 years (Table 1). The manifestations of the recurrent attacks are described in Table 2.

**Table 2:** Age and clinical characteristics of Rheumatic Fever patients with Sydenham's chorea at the first attack and recurrences presented (n = 17)

		*1^st^ attack*		*1^st^ recurrence*		*2^nd^ recurrence*		*3^rd^ recurrence*	
*Patient*	*Gender*	*Age (years)*	*Jones Major Criteria*	*Age (years)*	*Jones Major Criteria*	*Age (years)*	*Jones Major Criteria*	*Age (years)*	*Jones Major Criteria*
*1*	*Fem.*	*12*	*co*	*13*	*co+ar*	*14.5*	*ar*		
*2*	*Male*	*4*	*co*	*4.7*	*co*				
*3*	*Fem.*	*12.6*	*co*	*13*	*co*				
*4*	*Fem.*	*11.9*	*co*	*12.3*	*co*	*12.6*	*co*		
*5*	*Fem.*	*6.9*	*co*	*7.2*	*co*				
*6*	*Fem.*	*11.1*	*co*	*13.2*	*ca*				
*7*	*Fem.*	*8.2*	*co*	*10.1*	*co*				
*8*	*Male*	*11.7*	*co*	*14.1*	*ca+ar*				
*9*	*Male*	*12.7*	*co+ca*	*13.1*	*co*				
*10*	*Male*	*11*	*co+ca+ar*	*15*	*co+ar*				
*11*	*Male*	*9.2*	*co+ar*	*9.9*	*ca+ar*				
*12*	*Male*	*9.3*	*co+ca+ar*	*10.8*	*ar*				
*13*	*Fem.*	*10.8*	*co+ca+ar*	*11.2*	*co*	*12.2*	*co*		
*14*	*Fem.*	*10.1*	*co+ar*	*10.6*	*ar*	*13*	*ar*	*14.3*	*co+ar*
*15*	*Fem.*	*9.4*	*co+ca*	*13*	*ar*				
*16*	*Fem.*	*7.6*	*co+ca+ar*	*15.6*	*co*				
*17*	*Male*	*9*	*co+ca*	*14.5*	*co+ar*				

***Fem. - female; co- chorea; ca- carditis; ar- arthritis.***

Of the 30 patients who presented with isolated chorea at the first attack, 8 (26.6%) had disease recurrence: 6 with chorea (5 in isolation and 1 in association); 1 with carditis; and 1 with carditis and arthritis. Of the 56 patients who presented with chorea associated with another major disease manifestation during the first attack, 9 (16%) had recurrences. The intervals between the attacks of chorea can also be found in Table 2.

According to the information from the patient or parents, 61 children (71%) had no failure in the secondary prophylaxis while 25 (29%) were not compliant.

Of the 17 patients who presented recurrences, 9 (53%) showed good compliance.

Anti-streptolysin O was measured in 8 out of 14 patients with disease recurrence and was found to be normal in all of them.

## DISCUSSION

Rheumatic fever is still a prevalent disease in our country and an important cause of morbidity due to chronic cardiopathy.^9^

Sydenham's chorea was described by Thomas Sydenham in 1686 and it has been associated with rheumatic fever since 1956.^10^ It is a major manifestation of rheumatic fever and, according to the latest modification of the Jones criteria in 1992, a criterion sufficient even by itself for the diagnosis of the disease.^8^ The incidence of Sydenham's chorea has been increasing in our country over the last few years, as demonstrated in a recent publication.^1^ In a study undertaken in 7 centers in the State of São Paulo (Brazil) with 786 patients, chorea was present in 38% of rheumatic fever patients (299 patients).^11^

In our study, Sydenham's chorea occurred in 30% of our 290 patients (86 patients), which is similar to the frequency found by Cardoso et al,^12^ but higher than the frequency reported in the majority of the studies.^8,13^ Interestingly, of the 86 patients who presented with chorea at the first attack, approximately 60% had carditis in association with it. However, we did not observe a predominance of isolated chorea, as described in the literature^14^. We speculate that this fact may be related to the referral practice among our patients, where some of them, especially those with isolated chorea, are followed up by other specialists. Additional studies are necessary to confirm whether this fact may also be related to specific characteristics of our patient population or even to the streptococcal strains involved.

Nevertheless, of the 22 recurrences, only 3 (13.6%) presented isolated or associated carditis. Thus, we did not detect a higher probability of recurrences with cardiac involvement in patients who presented chorea at the first attack, as described in the literature.^15^ Cardoso et al. have suggested that carditis could be related to longer duration for Sydenham's chorea.^16^

Overall, 17 children (19.7%) presented with recurrences; of these, 9 used regular prophylaxis and 8 were non-compliant. Since we were unable to determine serum levels of penicillin in these patients, compliance was assessed according to the information from patients and parents.

In 14 out of 22 recurrences, chorea was present, either in isolation or in association with another manifestation. We did observe a predominance of chorea (63%) in the recurrences, which proves the tendency of chorea to mimic its first attack.^17^

Since we were intrigued by the high frequency of recurrences in patients with chorea who were on regular prophylaxis, we would like to emphasize a few issues:

1. We believe that the recurrences, including those in patients with a short interval (4 months) between the attacks, are not a continuation of the same attack, since those children remained asymptomatic and did not receive any treatment for their chorea during this period. It is important to mention that there is no reference in the literature regarding the minimum time required between two attacks;
2. There is a possibility that a chorea attack may be induced by non-streptococcal stimuli other than those already known (birth-control pills and pregnancy), which could explain the recurrences in patients receiving regular prophylaxis;
3. The efficacy of the antibiotics used in secondary prophylaxis may be limited due to the uncertain quality of some products;
4. Other non-rheumatic causes of chorea, such as viral infections, tumors of the central nervous system, degenerative processes, among others,^18-21^ have to be considered. This is especially so for those patients who present with recurrent isolated chorea while on adequate prophylaxis.

There is a lack of clinical and laboratory evidence for prior streptococcal infection and a lack of elevation of acute-phase proteins in the great majority of Sydenham's chorea patients. This leads us to believe that pure chorea is a "special" manifestation of rheumatic fever or that, in some cases, it may be related to another diagnosis. In some individuals, the recurrence of rheumatic fever is caused by light or transient streptococcal infections that may remain undetected in culturing or immunological tests.^7^ In this study, we did not observe a significant increase in anti-streptolysin O titers in any patient.

Kulkarni et al. described a 21% relapse rate in cases of Sydenham's chorea. However, data on the clinical characteristics of the patients or their prophylaxis compliance are lacking.^14^

In the opinion of Berrios et al., some of these chorea episodes do not represent distinct attacks but exacerbations in patients who have chronic and persistent choreic activity, characterizing the natural course of the disease.^7^ We believe, however, that after a minimum period of 4 months without clinical manifestations *or specific treatment*, the recurrence of choreic movements represents a clinical relapse rather than an ongoing disease.

## CONCLUSION

Despite the regular use of secondary benzathine penicillin prophylaxis, children with rheumatic fever are at a high risk for the recurrence of Sydenham's chorea.

Since the relationship of chorea and its recurrences to other clinical manifestations of rheumatic fever is poorly understood, rigorous prophylaxis with penicillin and periodic follow-up are necessary.
